# Normothermic Mouse Functional MRI of Acute Focal Thermostimulation for Probing Nociception

**DOI:** 10.1038/srep17230

**Published:** 2016-01-29

**Authors:** Henning Matthias Reimann, Jan Hentschel, Jaroslav Marek, Till Huelnhagen, Mihail Todiras, Stefanie Kox, Sonia Waiczies, Russ Hodge, Michael Bader, Andreas Pohlmann, Thoralf Niendorf

**Affiliations:** 1 Berlin Ultrahigh Field Facility (B.U.F.F.), Max Delbrueck Center for Molecular Medicine, Berlin, Germany; 2 Max Delbrueck Center for Molecular Medicine, Berlin, Germany; 3 Experimental and Clinical Research Center, a joint cooperation between the Charité Medical Faculty and the Max Delbrueck Center for Molecular Medicine, Berlin, Germany

## Abstract

Combining mouse genomics and *functional magnetic resonance imaging* (fMRI) provides a promising tool to unravel the molecular mechanisms of chronic pain. Probing murine nociception via the *blood oxygenation level-dependent* (BOLD) effect is still challenging due to methodological constraints. Here we report on the reproducible application of acute noxious heat stimuli to examine the feasibility and limitations of functional brain mapping for central pain processing in mice. Recent technical and procedural advances were applied for enhanced BOLD signal detection and a tight control of physiological parameters. The latter includes the development of a novel mouse cradle designed to maintain whole-body normothermia in anesthetized mice during fMRI in a way that reflects the thermal status of awake, resting mice. Applying mild noxious heat stimuli to wildtype mice resulted in highly significant BOLD patterns in anatomical brain structures forming the *pain matrix*, which comprise temporal signal intensity changes of up to 6% magnitude. We also observed sub-threshold correlation patterns in large areas of the brain, as well as alterations in mean arterial blood pressure (MABP) in response to the applied stimulus.

The treatment of chronic pain remains a major clinical challenge. Unraveling the mechanisms of central pain processing requires the investigation of large-scale brain circuits^1^,^2^. Magnetic resonance imaging (MRI) permits functional brain mapping via the *blood oxygenation level-dependent* (BOLD) effect^3^ and allows a direct comparison of human and animal data^1^,^2^. Combining functional MRI (fMRI) with mouse genetics offers a unique potential for non-invasive studies of endogenous proteins in nociceptive transduction pathways, central processing and pathogenesis^4^,^5^,^6^,^7^. Yet fMRI’s full potential for research into murine pain processing remains largely untapped, mostly due to methodological constraints^4^,^5^.

Recent technical and procedural developments in mouse fMRI with subcutaneous electrostimulation have achieved BOLD sensitivities comparable to human fMRI^8^,^9^,^10^,^11^. However, subcutaneous electrostimulation is inherently non-physiological and randomly excites proximate neurons of various somatosensory modalities^6^. Cutaneous thermal stimulation, on the other hand, permits the study of distinct somatosensory transduction pathways with respect to their thermal activation thresholds^7^. Pioneering studies have demonstrated the proof-of-principle for mouse fMRI with nociceptive heat stimuli^12^,^13^,^14^. Yet in these studies, the BOLD sensitivity was an order of magnitude below that reported for electrostimulation, putting it at the borderline of detection levels. This hinders a detailed investigation of endogenous target proteins in murine pain processing.

Here we combine previous technical and procedural accomplishments in mouse fMRI^8^,^9^,^10^,^11^,^15^ with heat stimulation to probe central nociception in mice. In light of the physiological side effects of general anesthesia, we attuned our setup to improve the maintenance of murine physiology under experimental conditions. Our method includes (i) a mouse cradle that generates uniform and physiological body temperatures in anesthetized mice, (ii) an MRI-compatible stimulation device permitting controlled thermal stimulation, (iii) the application of a high sensitivity *radio-frequency* (RF) probe^8^, and (iv) data processing tools to enhance the detection of the BOLD signal. We also monitored the mean arterial blood pressure (MABP), since cardiovascular alterations in response to acute stimuli have been reported to constitute a potential confounder for hemodynamic readouts in small animals^16^,^17^,^18^,^19^,^20^.

When applied to wild type BL6 mice, we achieved spatially discrete BOLD effects of up to 6% magnitude for mild noxious 46 °C stimuli at the plantar hindpaw. The precise influence of MABP on the BOLD signal remains to be determined.

## Results

### Towards physiological body temperatures in mouse MRI

Under general anesthesia, mice become poikilothermic^21^,^22^. To study physiological processes such as nociception using fMRI, an animal’s body temperature ought to be maintained at physiological thermal conditions. Artificial warming of anesthetized mice aims to preserve body temperatures within the *limits of normothermia* (LiN)^22^,^23^. Commonly in mouse fMRI, floor heating ensures a rectal temperature range of 35.5–37.5 °C^8^,^9^,^10^,^11^,^24^, but its effect on body surface temperatures has not yet been evaluated.

**Figure 1:**
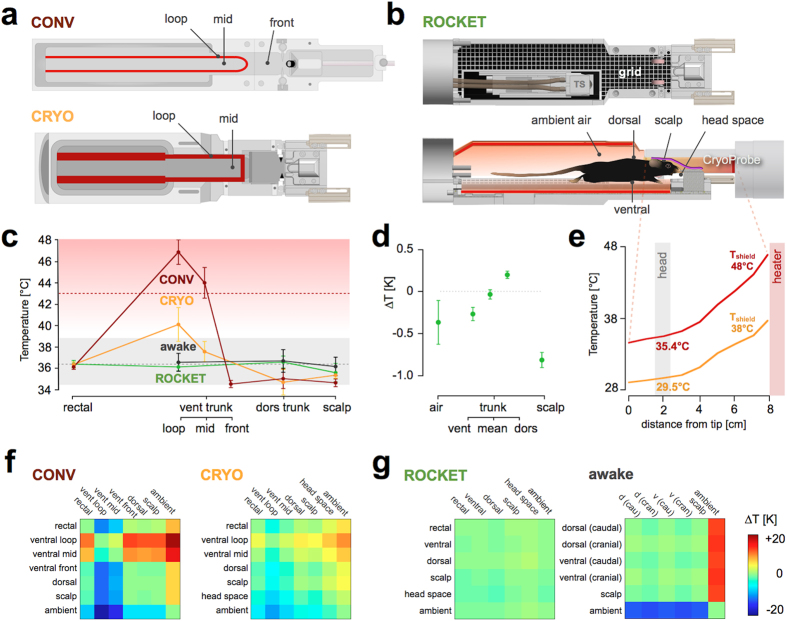
Thermal environments. **(a)** Schematic of the conventional head radio frequency coil (CONV) and CryoProbe (CRYO) mouse cradles (view from above) illustrating their heating system – a warm water circulation loop (red) – and indicating the ventral temperature measurement sites (loop, mid, front). **(b)** Schematic of the ROCKET setup (top: view from above; bottom: view from side) illustrating geometry and temperature measurement locations for ambient air temperature, dorsal and ventral trunk, scalp temperature and ambient air temperature around the head (head space) in all setups. The suspended grid for placing the animal allows for non-contact warming of the animal. The insulating sheet between head and coil surface is illustrated in magenta. The thermostimulation system (TS) is shown from above in the top panel. **(c)** Body temperatures of awake (n = 9) and isoflurane anesthetized mice in three fMRI setups: CONV (n = 5), CRYO (n = 5), and ROCKET (n = 6) setup. Rectal temperature and body surface temperatures – scalp, dorsal and ventral trunk at different locations (loop, middle (mid) and front) – were plotted (mean (s.d.)); see measuring locations in panels (**a,b**). Range of circadian CBT variation (gray shading); temporal mean (gray dotted line)^23^. The red gradient indicates cumulative equivalent minutes at 43 °C (red dotted line) beginning at 39 °C^27^. **(d)** Temperature differences of respective body sites referenced against rectal temperature measured in the ROCKET (mean ± s.e.m.). **(e)** Thermal gradient of the CryoProbe surface with thermal shield set to 38 °C and 48 °C (measured outside the scanner at room temperature (20 °C)). Note the marked increase in surface temperature towards the internal heater located 8 cm distal from experimental head position (see (**b**)). **(f,g)** Color-coded temperature difference matrices illustrate body temperature distribution (at 36.3 °C ± 0.5 °C rectal temperature) within the tested experimental setups versus awake mice. The ROCKET setup resembles the situation of awake mice, except for the ambient air temperature, which must be raised to CBT in the ROCKET setup. **(a–g)** Ambient air temperatures were 20.0 °C (awake), 28.2 °C (CONV), 30.6 °C (CRYO) and 36.0 °C (ROCKET).

Here we provide a detailed comparison of the thermal condition of anesthetized mice in two experimental MRI setups: i) a common animal cradle customized for a conventional mouse head RF coil array (CONV), and ii) a cradle tailored for a state-of-the-art high sensitivity mouse head RF coil (*CryoProbe*, CRYO) (Fig. 1a). For mice anesthetized using isoflurane, the cradle’s heating system was adjusted to sustain a rectal temperature of 36.3 °C ± 0.5 °C while the spatial temperature distribution across the skin was monitored. The floor heating system of the two setups consisted of a loop of integrated pipes supplying pre-heated water (Fig. 1a). As a control, the skin temperatures of awake mice were measured outside of the MR suite at room temperature (20 °C). The dorsal and ventral trunk yielded temperatures of 36.7 °C (1.38 °C) and 36.6 °C (1.51 °C) (mean (s.d.)). The scalp temperature was slightly lower, at 36.2 °C (0.88 °C) (Fig. 1c).

Mice positioned in the CONV cradle exhibited skin-floor-interface temperatures in proximity to the floor heating that lay well above the LiN (*loop* position 47.0 °C (1.17 °C), *mid* position 44.1 °C (1.46 °C); Fig. 1a,c). More distal temperatures at the *front* position dropped below the rectal temperature (34.5 °C (0.34 °C), 36.1 °C (0.23 °C)). Skin temperatures at the dorsal trunk and the scalp (34.6 °C (0.36 °C), 35.0 °C (0.94 °C)) were slightly closer to the rectal temperature; Fig. 1c)). To achieve greater accuracy for all scalp temperature measurements, a thin insulating sheet was introduced between the fiber optic temperature probe and the RF coil surface (Fig. 1b; Online Methods). The ambient air temperature was 28.2 °C (2.65 °C).

Mice placed in the CRYO cradle yielded temperatures that were more evenly distributed across the skin (Fig. 1c). The CryoProbe is surrounded by a heated thermal shield which controls the coil surface temperature^8^. The manufacturer’s setting of 38 °C for the thermal shield temperature was used. Skin-floor-interface temperatures at the *loop* position (40.1 °C (1.6 °C)) deviated less from the LiN than the values for animals in the CONV cradle. Temperatures at the *mid* position of the ventral trunk (37.6 °C (0.96 °C)), dorsal trunk (34.7 °C (1.20 °C) and scalp (35.3 °C (0.29 °C)) varied around the rectal temperature (36.3 °C (0.25 °C)). The ambient air temperature for the CRYO setup (30.6 °C (0.84 °C)) was higher than for the CONV setup (ΔT = 2.43 °C ± 1.23 °C; mean ± s.e.m.).

To achieve a uniform normothermic body temperature distribution for anesthetized mice placed in the MR bore, we developed a novel setup that provides convective, non-contact body warming: a mouse bed customized for the geometry of the CryoProbe setup, which we call the *ROdent Convective Keg-shaped Environment for Thermostimulation (ROCKET)* (Fig. 1b; Online Methods). The ROCKET contains a thermal chamber that surrounds the trunk and tail of the mouse. The animal’s body resides on a plastic grid suspended between the chamber’s walls, which are heated by warm water circulation (Fig. 1b). This creates a heat transfer driven predominantly by convection and radiation. An independent temperature management of the ROCKET’s upper and lower sections creates a thermal equilibrium accompanied by uniform body surface temperatures. An ambient air temperature of 36.0 °C (0.62 °C) produced a rectal temperature of 36.4 °C (0.08 °C), a ventral temperature of 36.6 °C (0.21 °C), and 36.1 °C (0.16 °C) for the dorsal trunk (Fig. 1c). ROCKET rectal temperatures were consistent with trunk temperatures (dorsal, ΔT = 0.20 °C ± 0.07 °C; ventral ΔT = 0.27 °C ± 0.09 °C; dorsal/ventral mean, ΔT = 0.03 °C ± 0.14 °C) and with the ambient temperature (ΔT = 0.36 °C ± 0.26 °C) (Fig. 1c,d).

To achieve a more physiological scalp temperature, the CryoProbe’s thermal shield setting was adjusted to 48 °C. Notably, the coil surface temperature at the mouse head position was significantly lower than the shield temperature (Fig. 1e). This approach resulted in a scalp temperature of 35.6 °C (0.23 °C) and permitted a slight reduction of the difference between scalp and rectal temperatures of ΔT_scalp-rectal_ = 0.81 °C ± 0.09 °C versus ΔT_scalp-rectal_ = 1.0 °C ± 0.16 °C for the CRYO setup (with thermal shield adjusted to 38 °C). The ambient temperature around the head was 0.40 °C ± 0.36 °C below the scalp temperature and 0.8 °C ± 0.43 °C below the ambient temperature measured above the trunk.

In contrast to the CONV and CRYO setup (Fig. 1f), the temperature homogeneity of anesthetized animals placed in the ROCKET compared remarkably well with that of awake mice (Fig. 1g).

### Thermostimulation unit within the ROCKET

**Figure 2:**
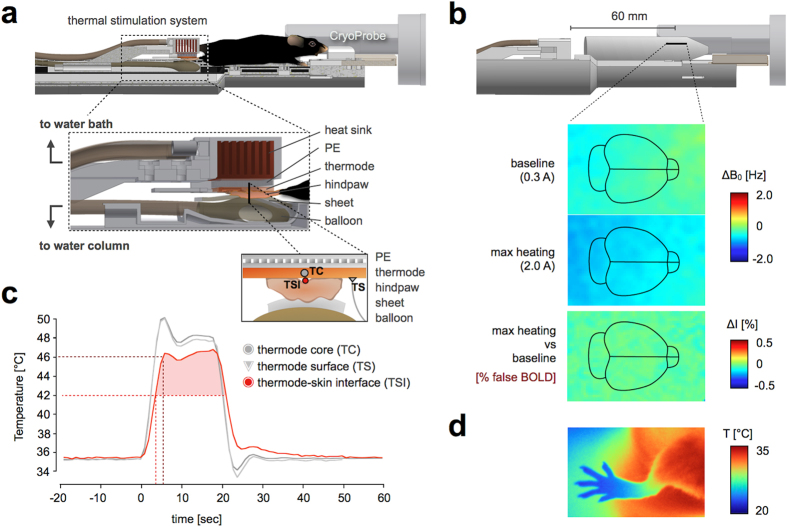
Thermostimulation setup. **(a)** Top panel: Implementation of the thermal stimulation device for the plantar hindpaw within the ROCKET mouse holder system. Bottom panel: Functional scheme of the thermal stimulation system: the Peltier element (PE) is attached to a heat sink cooled by water circulation. The PE’s driving current is feedback controlled, and monitored by a thermoprobe within a copper plate (the thermode) covering the entire PE surface. The paw is positioned between the thermode and an inflatable balloon connected to a water-filled cylinder, whose filling level determines the contact pressure. **(b)** Top panel: To test for B_0_-effects induced by the stimulation system a 2% agarose phantom was positioned below the CryoProbe. Bottom panel: Coronal views of field changes measured in an agarose phantom induced by the current in the Peltier element. Top: Field change induced by baseline heating current; center: Field change induced by maximum heating current; bottom: Expected signal change in percent due to field change induced by switching from baseline heating to maximum heating. The contours indicate the position of the mouse brain during fMRI. **(c)** Stimulation temperature plot during a defined heat paradigm (35.5 °C baseline, 48 °C stimulus, 4 °C/s, 20 s). Measuring locations are illustrated in the above scheme: the PE control temperature measured within the thermode core (TC), on the thermode surface (TS) and at the thermode-skin-interface (TSI). Dashed lines indicate the delays until TSI temperature reaches 42 °C (TRPV1 activation threshold) and 46 °C (stimulation target temperature). **(d)** Infrared image of a hindpaw from an awake mouse indicating its adaptation to the respective thermal ambient. Measured at room temperature (20 °C).

To apply focal thermal stimulation in a controlled and reproducible way, we used ramped contact heat transfer based on a water-cooled, feedback-controlled Peltier element with a copper plate as a contact thermode. The hindpaw was positioned between the thermode and a thermally insulating sheet that lay on an inflatable hydraulic balloon (Fig. 2a). The balloon was connected to a water-filled cylinder, whose filling level ensured a defined, even pressure between the uneven surface of the paw and the thermode (Online Methods). This permits applications of thermal stimuli to the plantar surface of the animal’s hindpaw (for dorsal stimulation see Supplementary Fig. S6d online).

Prior to the experiments, we assessed the MRI compatibility of the thermostimulation device to rule out any potential influence of electric currents from the Peltier element on the magnetic field (B_0_) homogeneity, which could lead to false BOLD activations. In the “worst case scenario”, when heating rose from baseline to maximum, the absolute signal change in MR image intensity (ΔI) was estimated at less than 0.1% for the region of interest: the location of the mouse brain in the apparatus during an fMRI study (Fig. 2b; Online Methods). To put this into perspective, the typical temporal signal-to-noise ratio (tSNR) of the fMRI data in the cortex was 193, which corresponds to a level of noise in the fMRI signal-time courses of 0.58% (relative temporal standard deviation).

The stimulation paradigm was governed by the temperature of the contact thermode core (TC) (Fig. 2c), which was used as a feedback temperature for the computer-driven Peltier element (Online Methods). In contrast to the furry trunk and head, we observed that paw skin temperatures in awake mice adapt to their thermal environment (Fig. 2d). We therefore set the baseline temperature to 35.5 °C, to match the thermal chamber temperature (see Discussion). Baseline temperature oscillations at the paw did not exceed ±0.2 °C.

Differences between temperatures at the TC site and at the target thermoreceptor level must be taken into consideration in a thermostimulation paradigm. We also measured temperatures at the thermode’s surface (TS) and at the thermode-skin-interface (TSI), which reflects actual stimulation temperatures (Fig. 2c). While TC and TS were almost identical (∆T_max_ = 0.2 °C), comprising maximum ‘plateau’ temperatures of 48 °C (TC) and 47.8 °C (TS), the more relevant TSI revealed temperatures around 46 °C, without the initial overshoot observed in TC and TS. TSI displayed a significantly lower ascending temperature slope (2.5 °C/s) than TC or TS (both 4.0 °C/s).

The drop in temperature from thermode-core to thermode-skin-interface (Fig. 2c), more pronounced in the initial stimulation phase, may be attributed to the heat sink effect of the skin^25^. Skin thermal conductivity is low enough that peak temperature and transition rate might be further decreased at the thermoreceptor level^26^. To avoid tissue damage^27^, the total stimulus duration was limited to 20 s using a 14 s temperature plateau (Fig. 2c); no swelling or reddening of the skin was observed.

### fMRI feasibility study

**Figure 3:**
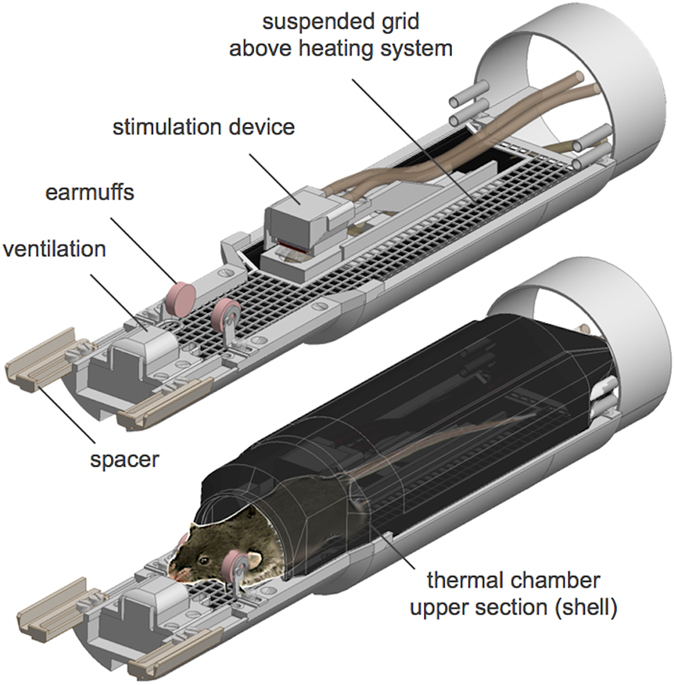
Quick overview of the ROCKET equipment. Top panel shows the suspended grid for animal positioning above the floor heating (detailed in Fig. 1), the thermal stimulation device for the plantar hindpaw (detailed in Fig. 2), earmuffs for head fixation and hearing protection (combined with earplugs, Online Methods), the ventilation block with a small bail (serving as a ‘tooth bar’ for rostral head fixation) and spacer to position the cradle relative to the transmitting /receiving section of the CryoProbe (head position, Fig. 1b). Bottom panel shows the positioning of the mouse on the suspended grid covered by the thermal chamber’s shell. The thermal chamber is completed by enclosure of the ROCKET’s frontal part with the CryoProbe (Fig. 1b).

**Figure 4:**
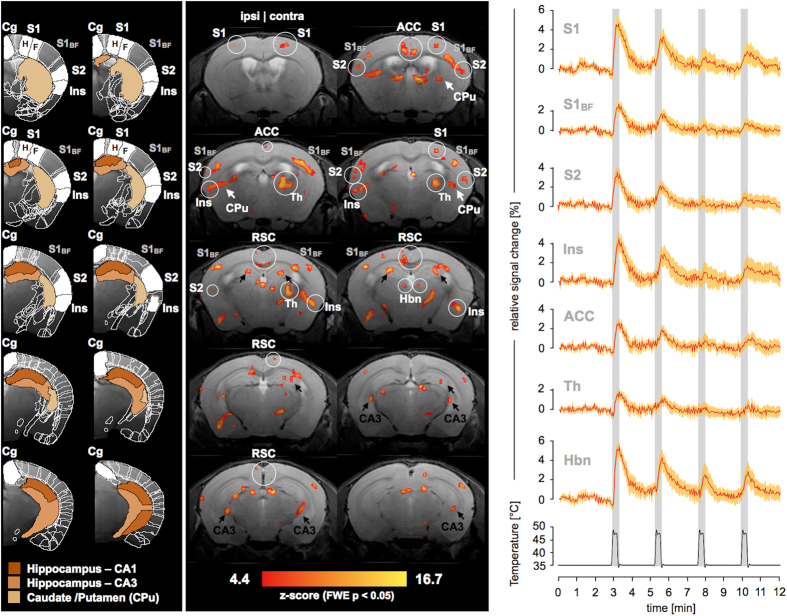
BOLD activation maps. Left panel: Anatomical mouse brain atlas in Waxholm space (WHS) superimposed on a high-resolution reference model. Structural elements of the *pain matrix* are displayed in white: the paw region of the primary somatosensory cortex (S1) composed of areas representing hindpaw (H) and forepaw (F), the barrel field area of S1 (S1_BF_), cingulate cortex (Cg), secondary somatosensory cortex (S2), and insular cortex (Ins). Hippocampal subfields CA1 and CA3, and caudate /putamen (CPu) are colored respectively (see color legend). Partial overlaps of anatomical regions result from maximum intensity projection during dimensionality reduction in z-direction (see Online Methods). Mid panel: Z-statistic parametric map from a mixed effects analysis of 6 mice in response to heat stimulation of the plantar hindpaw (FWE, p < 0.05) projected onto a structural reference scan. Significant clusters were found i.a. in structures of the *pain matrix* including the cingulate cortex (subdivided in anterior cingulate cortex (ACC) and retrosplenial cortex (RSC)), S1, S1_BF_, S2, thalamus (Th) including the habenula (Hbn), and the insular cortex (Ins). Arrows are used to indicate significant clusters in hippocampus (black) and CPu (white). Ipsi- and contralateral sides are labeled above. Right panel: Relative signal intensity change over time for significant voxels within defined anatomical brain regions (mean ± 2 s.e.m.). ROIs were defined based on significant clusters, which were masked by selected anatomical structures (see Online Methods).

This approach for the thermostimulation of normothermic mice was next examined in an *in vivo* feasibility study. Mice were anesthetized using isoflurane and placed in the ROCKET (Fig. 3), ensuring a uniform body temperature within the LiN. Animals were mechanically ventilated to maintain blood gas levels (tcpCO_2_) within the physiological range (20 to 50 mmHg)^28^, comprising a mean of 34.3 mmHg (2.4 mmHg) as determined in an initial experiment using a transcutaneous blood gas analyzer (Supplementary Fig. S1 online). A neuromuscular blocking agent was administered to prevent motion artifacts and block the animal’s normal withdrawal reflex upon a thermal stimulus^9^,^15^,^29^. The head was stereotactically fixed by replacing conventional ear bars with memory foam earmuffs, which attenuate acoustic noise caused by magnetic gradient switching during fMRI (Supplementary Fig. S5 online; Online Methods). The right hindpaw was attached to the thermode (Fig. 2a) and stimulated four times per fMRI session using TC = 48 °C, corresponding to a thermode-skin-interface temperature of approx. 46 °C (Fig. 2c, Fig. 4 right panel, bottom).

fMRI was conducted in six mice using a 9.4 Tesla small-animal MR system equipped with the ROCKET and a high sensitivity RF coil (CryoProbe). fMRI data were corrected for head motion and spatially smoothed. Global signal intensity drifts, respiratory induced B_0_ shifts and remaining head motion were identified by *independent component analysis* (ICA) and removed prior to statistical analysis (Supplementary Fig. S8 online; Online Methods). Data were distortion-corrected and normalized to an anatomical reference in Waxholm space^30^. A general linear model (GLM) was applied to each time-series and a mixed effects analysis was conducted for higher-level modeling using full FMRIB’s local analysis of mixed effects (FLAME, stages 1 + 2) to account for the small number of subjects^31^.

Statistical inference using *family-wise error* (FWE) correction at p < 0.05 (Z > 4.4) revealed distinct, highly significant BOLD patterns in several cortical and subcortical anatomical areas (Fig. 4, left panel). Significance patterns were primarily observed in the primary and secondary somatosensory cortex (S1 and S2), the thalamus (Th) and particularly the habenula (Hbn), insular cortex (Ins), the cingulate cortex (Cg) including anterior cingulate cortex (ACC) and retrosplenial cortex (RSC), hippocampus (CA1 and CA3), and the basal ganglia (caudate/putamen, CPu). Most structures were bilaterally activated, while the thalamus and S1 showed a strong contralateral preference. Tracking the temporal signal evolution for significant regions in anatomically defined brain areas showed prominent BOLD signal changes; e.g. as large as 6% in S1 (paw region) and the habenula or 3% and 5% in the anterior cingulate and insular cortex, respectively (Fig. 4, right panel). In all areas that were plotted, the magnitude of the signal declined for subsequent stimulation periods (Fig. 4, right panel).

**Figure 5:**
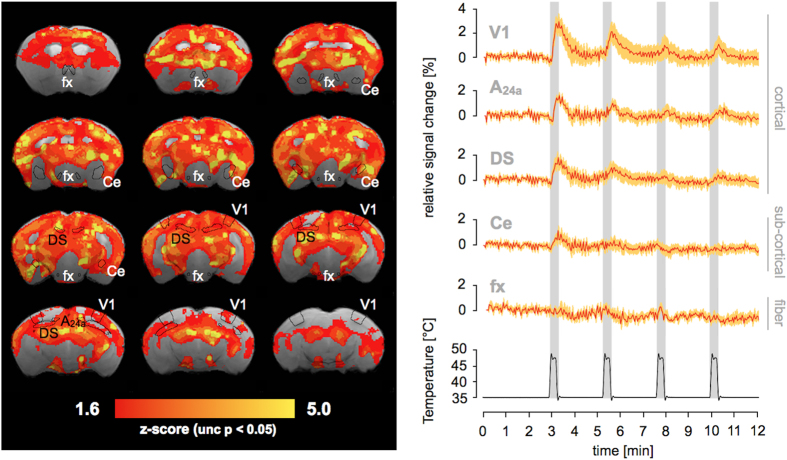
Statistical parametric map for uncorrected z-values. Left panel: Z-statistic parametric map from a mixed effects analysis of 6 mice in response to heat stimulation of the plantar hindpaw (p < 0.05, not corrected for multiple comparisons). The map shows significance patterns (below the statistical threshold shown in Fig. 4) in large areas of the brain. Anatomical areas, which were used as ROIs for signal intensity extraction are contoured. Right panel: Relative signal intensity change over time for defined anatomical brain regions (mean ± 2 s.e.m.). Plotted cortical structures are primary visual cortex, monocular area (V1), cingulate cortex, area 24a (A24a), and dorsal subiculum (DS). Plotted subcortical structures are central amygdaloid nucleus (Ce), and fornix (fx), which is a fiber group.

Statistical maps for uncorrected thresholds at p < 0.05 (Z > 1.6) yielded large significance clusters spanning multiple anatomical regions, which were non-significant with respect to the FWE corrected threshold (Fig. 5). Temporal signal changes for these regions revealed stimulus-correlations of lower magnitude, which were particularly pronounced in the cortex (Fig. 5, right panel).

**Figure 6:**
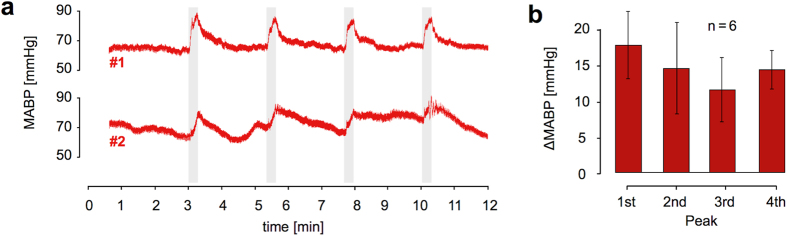
Cardiovascular monitoring. **(a)** Mean arterial blood pressure (MABP) measurements plotted for two representative mice. Animal #2 displays comparably unstable blood pressure. Both animals show an increase of MABP in response to subsequent heat stimuli. **(b)** Mean increase of MABP in six animals for each subsequent heat stimulus (mean ± sd).

In a separate study with six mice using the same setup outside the MR scanner, mean arterial blood pressure (MABP) was monitored to examine cardiovascular changes induced by the stimulus. The four subsequent heat stimuli were companied by increases in MABP of 17.9 ± 4.8 mmHg, 14.7 ± 6.5 mmHg, 11.7 ± 4.5 mmHg and 14.5 ± 2.7 mmHg, respectively (Fig. 6).

## Discussion

Much of our understanding of the human central nervous system comes from studies of animals in experimental settings. Drawing appropriate conclusions from these studies depends on the extent to which the settings reflect physiological conditions. This is difficult to achieve in murine imaging studies with noxious stimuli, where general anesthesia must be applied for ethical and practical reasons^4^,^5^.

Ideally, physiological and particularly thermoreceptive studies should be performed within the *thermal neutral zone* (TNZ), the “range of ambient temperatures where metabolic rate is at basal or resting levels”^23^. TNZ in awake resting mice lies in a particularly narrow region around 31 ± 1 °C^23^. General anesthesia renders mice poikilothermic; i.e., their body temperatures adapt to the thermal environment^15^,^16^. Solving this problem requires defining the *limits of normothermia* (LiN)^23^ in awake mice and providing a method to replicate body temperatures within these limits during experiments.

The *core body temperature* (CBT) of awake C57BL/6 mice fluctuates between approx. 34.5 °C and 38.5 °C, depending on their activity and the phase of their circadian cycle^23^. While this range might be considered as normothermic, the average CBT of approx. 36.3 °C^23^,^32^ corresponds to that of awake, resting animals within the TNZ^32^. Subcutaneous temperatures of the dorsal trunk were previously shown to closely resemble those of the body core^33^,^34^. We found that the skin temperature across furry body parts (such as the scalp and trunk) is uniformly distributed at about 36.5 °C in awake mice (Fig. 1c,g), with a maximum within-subject temperature difference of 1.2 °C ± 0.19 °C (Fig. 1g; Supplementary Fig. S2 online). We conclude that adjusting the overall body temperature to 36.3 °C yields physiological skin and core temperatures that most closely correspond to those of awake, resting mice.

To maintain an overall physiological, uniform body temperature under experimental conditions, we found that it is essential i) to provide a homogeneous ambient temperature and ii) to raise the environmental temperature to the desired CBT – resulting in strikingly similar temperatures for rectal and cutaneous sites (Fig. 1d,g). By developing a novel customized mouse cradle design (ROCKET; Fig. 1b), which allows for a controlled, uniform warming of the anesthetized mouse, it was possible to closely reproduce the thermal conditions of awake mice within the MR system (Fig. 1c,d,g).

In contrast, non-uniform partial warming of the trunk might produce normothermic rectal temperatures but cannot ensure similar physiological temperatures across the body surface and the true body core, which includes the heart, spinal cord, and the brain. We found that a physiological rectal temperature can be maintained by warming a comparably small fraction of the trunk when heating is raised to a temperature far above the normothermic range. In the CONV setup, skin temperatures above the heating loops were as high as the applied noxious stimulus (up to 47 °C), a situation which might cause a thermal bias inappropriate to studies of nociception^35^. Persistent heat stimuli were shown to mask the perception of transient noxious stimuli at extrasegmental body sites – a phenomenon known as diffuse noxious inhibitory control^35^,^36^,^37^,^38^,^39^,^40^. Ambient and body temperature have an impact on various physiological processes, thermoreception and pain^35^,^41^,^42^.

The CRYO setup (Fig. 1a) represents a major improvement towards making the thermal state of the animal’s body more homogeneous and physiological^43^. It permits floor temperatures to be significantly reduced (Fig. 1c,f), but skin temperatures above the heating loops still exceed those of awake mice under physiological conditions. The ROCKET affords skin temperatures that uniformly correspond to the CBT, which is at the very least an essential reference for assessing thermal conditions in other mouse MRI setups.

Yet there is a lack of studies which aim to investigate the influence of murine body temperatures on somatosensation and nociception using hemodynamic readouts. The ROCKET was developed to preserve physiological thermal conditions of awake mice. This may not be reflected in higher BOLD magnitudes. Mild hypothermia of the scalp has been reported to significantly increase BOLD amplitudes in mice for somatosensory stimulation due to vascular effects^8^.

In addition to affording a non-contact, convective heating system, the modular design of the ROCKET provides a spatially adaptable stimulation unit (Fig. 2a; Supplementary Fig. S6 online). A circulating-water-enhanced Peltier element permits the rapid and controlled application of precise heat and cold stimuli^25^. The stimulation device is relatively close to the brain (compared to rat fMRI setups), making it necessary to establish that the device does not interfere with the MR signal, and thus exclude stimulus-correlated false positive BOLD activations (Fig. 2b). Since the noise in the fMRI data is typically at least 5 times larger than the estimated maximum signal change induced by the Peltier element (0.58% versus <0.1% respectively), the effect of the stimulation device can be considered negligible.

Despite the controversy surrounding the benefit of thermode application force on thermal thresholds and pain^44^,^45^, applying mild contact pressure has a critical impact on the conformation of the thermode and the uneven surface of the paw. A uniform contact is required for reliable heat transfer^45^,^46^. In our setup, a dedicated inflatable hydraulic balloon connected to a water-filled cylinder ensures defined and reproducible contact pressures by the cylinder’s water column level and allows a comfortable positioning of the paw (Supplementary Fig. S6b online).

The baseline temperature of contact thermodes is known to influence thermal perception^42^,^45^,^46^. The thermal adaptability of the murine paw (Fig. 2d) makes it difficult to define a thermoneutral baseline temperature. Here we set the baseline temperature to approximate the overall skin temperature, which was adapted to maintain the LiN. This measure compares favorably with the preferred footpad temperature of awake animals within the TNZ^47^. Fluctuations in baseline temperature were held within the ±0.2 °C range so that known temperature reception thresholds were not exceeded^25^.

The thermal feedback control permits a tight regulation of the thermal transition rates, which have been reported to influence heat transfer and pain response^25^. Thermal stimuli of 46 °C were applied to exceed the thermal activation threshold of the moderate noxious heat receptor TRPV1^7^. Indeed, nociceptive Aδ-fibers were previously found to start firing in response to 42 ± 3 °C (and C-fibers at around 40 °C) in murine glabrous skin of the hindpaw *in vivo*^48^.

The *in vivo* application of our mouse fMRI methodology yielded spatially discrete BOLD effects of up to 6% magnitude for mild noxious stimuli of 46 °C (Fig. 4). Significant clusters were found in brain areas that agree with structures that are activated in response to noxious heat stimulation in humans^49^,^50^,^51^ and rats^4^,^5^,^52^, as identified in previous fMRI studies. Most of these structures are elements of the so called *pain matrix*^53^,^54^,^55^,^56^.

A predominantly contralateral activation was observed in the thalamus, which relays afferent information from the periphery to cortical and subcortical areas for further processing^57^. A strong contralateral activation was detected in the S1 subregion, which holds a somatotopic representation of the stimulated paw^58^. The activation of this area is expected for the processing of noxious and innoxious somatosensory inputs^56^. The barrel field region of S1 was found to be bilaterally activated with contralateral preference. This was previously observed in fMRI studies with rodents using acute stimuli^9^,^52^ and might be interpreted as an unspecific response to a salient event^53^,^54^,^55^,^56^. S2 is involved in the processing of the quality characteristics of somatosensory stimuli^25^. Activity in the anterior cingulate and retrosplenial cortex is expected for the processing of emotional aspects of pain^59^. The insular cortex plays a role in the maintenance of body integrity and pain modulation^50^. Highly significant activations were found in both symmetric nuclei of the habenula, a small posterior-medial aspect of the dorsal thalamus which is also involved in the modulation of pain^60^. We additionally found stimulus correlation patterns in the hippocampal subfields CA1 and CA3, and the basal ganglia (caudate/putamen, CPu). Hippocampal activation has been reported in studies of pain in rats^52^,^61^ and humans^62^, where it is thought to be involved in pain inhibition^63^. A key role in nociception has been attributed to CPu^64^.

Signal time courses of all anatomical areas showed a decline of maximum BOLD magnitudes for subsequent stimulation periods (Fig. 4, right panel). This has previously been observed in studies of electrostimulation of mice anesthetized with isoflurane^9^ and might be due to anesthetic effects (e.g. neural depletion)^65^, peripheral or central habituation. For heat stimuli, habituation was reported as discharge decrement at the fiber level (Aδ- and C-fiber nociceptors^66^,^67^,^68^) and receptor level (heat transduction channel TRPV1^69^,^70^). Central mechanisms have also been reported to contribute to habituation to heat pain^71^,^72^,^73^. Sensitization, as described for noxious stimulation^74^,^75^,^76^, was not observed, which appears to be related rather to tissue damage^26^,^72^,^77^.

Previous reports have shown that heat stimulation fMRI can be used to phenotype transgenic mouse models with regards to pain^12^,^13^,^14^. A comparison of our findings with these pioneering studies revealed differences in the data at similar stimulation temperatures, including a five- to tenfold increase in BOLD magnitude. The functional significance patterns observed in our study appear more specific to anatomical structures and differ in terms of spatial distribution^12^,^13^,^14^. BOLD patterns and magnitudes observed in our study accord with a just published report on mouse fMRI applying a comparable stimulation paradigm within the CRYO setup^43^. That study utilized focal laser stimulation (at 45–46 °C) and a similar animal preparation protocol including a neuromuscular blocking agent and mechanical ventilation.

To account for the multiple comparison problem, we applied statistical inference using family-wise error (FWE) correction at p < 0.05. Lowering the statistical threshold to uncorrected z-values revealed large significance patterns which span multiple anatomical regions (Fig. 5). In particular, cortical areas (e.g. the primary visual cortex or dorsal subiculum) showed remarkable magnitudes for the first stimulation period. Subcortical structures (e.g. the central amygdaloid nucleus) showed a lower correlation with the stimulus (Fig. 5, right panel).

A recent study comparing the effects of different types of anesthesia in mouse fMRI reported similar findings for electrostimulation paradigms^11^. Here the authors argued that the sub-threshold patterns that were observed might originate from stimulus-induced alterations in mean arterial blood pressure (MABP). Rapid changes in systemic MABP may not be sufficiently buffered by murine autoregulatory mechanisms and thus affect hemodynamic readouts such as cerebral blood volume (CBV), cerebral blood flow (CBF) and the BOLD response. Although MABP was not directly monitored in that study, changes in heart rate, pulse distention and O_2_ saturation were found to increase in correlation to the stimulus^11^.

Here we monitored MABP invasively and observed alterations of 7 to 25 mmHg in response to the stimulus. For some animals, MABP changes displayed similarities to the signal evolution of the hemodynamic response (Fig. 6). Previous work has shown that pharmacologically induced changes in MABP in this range do not affect CBV in rats anesthetized with halothane^16^ or α-chloralose^20^. However, MABP increases of 35 mmHg and above were found to induce region-specific hemodynamic responses which might be mistaken for neuronal activation^16^,^20^.

Yet a direct translation of these findings is difficult: The species under investigation and the anesthetic regime that is applied might have an influence on the ability of the cerebral vasculature to maintain CBF unaffected when cardiovascular alterations arise^78^,^79^. The operational window under the above conditions is described as a MABP range of 100–120 mmHg^16^,^20^. In isoflurane-anesthetized mice the MABP baseline is shifted to lower values^80^, leading to a detection of alterations in the range of 70–90 mmHg (Fig. 6a). Since the efficiency of autoregulation further depends on the transition rate of MABP alterations^17^,^18^, an application of ramped heat stimuli constitute another challenge when attempting to translate the findings described above to our data.

Assuming that MABP constitutes a confounding factor for the BOLD signal, statistical inference would probably not be sufficient to account for it. Some brain areas have been shown to be more susceptible to cerebral blood pressure alterations than others, which would translate into specific BOLD patterns in the brain^16^,^20^. Yet it appears unlikely that the significance pattern resembling the *pain matrix* that we observed is exclusively evoked by changes in MABP: Significance patterns which appear contralateral for elements of the spinothalamic pathway (thalamus, S1), symmetrical for structures such as the habenula and bilateral for other components of the *pain matrix* are in agreement with previous data in humans^25^,^49^,^50^ and rats^4^,^5^,^52^.

The impact of MABP remains uncertain. Its potential implications for the general feasibility of mouse fMRI studies using acute nociceptive (and innoxious^11^) stimuli demands further investigation. It would be instructive to evaluate the origin of the observed arousal by simultaneous monitoring of BOLD and MABP^15^,^16^. A strong correlation between both measurements might constitute a plausible indicator for a confounding effect. However, systemic alterations in MABP are expected as responses to acute and nociceptive stimuli, which might be sufficiently buffered by autoregulation, whereas a neural origin of the observed arousal could not be excluded. Since the animals are only lightly anesthetized, a sudden stimulus may trigger an arousing or alerting response predominantly in cortical areas. Consequently, techniques for the direct measurement of neural activity need to be included to ascertain the origin of the arousal and the effective influence of MABP on the BOLD signal in anesthetized mice.

In summary, we show that heat stimulation in mouse fMRI can achieve BOLD magnitudes that are comparable to those reported for electrostimulation in previous studies^8^,^9^,^10^,^11^, but it also faces similar challenges^11^. The present approach substantially enhances BOLD sensitivity for heat stimuli by building upon technical and procedural accomplishments demonstrated in recent mouse fMRI studies^8^,^9^,^10^,^11^,^15^ and combining these with novel developments to create a more physiological, reliable mouse fMRI. The advances of our method include a) maintenance of whole-body physiological temperatures, b) an MR-compatible, feedback-controlled thermal stimulation system with c) reliable contact pressure, d) choice of a large thermode surface to achieve spatial summation by covering the receptive fields of multiple nociceptors^81^, e) hearing protection to reduce acoustic stress^4^,^82^, f) independent component analysis-based signal filtering to eliminate respiration and hardware-related signal components that impair BOLD detection^83^, and g) distortion correction of functional images acquired at ultrahigh field strength^84^, combined with an accurate spatial normalization algorithm^85^,^86^ to facilitate detection of consistent BOLD patterns at a group level. Established measures included boosting the signal-to-noise ratio by employing a state-of-the-art high sensitivity RF coil for signal transmission/reception^8^ and controlling physiological and motion parameters with a muscle relaxant in conjunction with mechanical ventilation^9^,^15^,^29^.

This method achieves a major improvement over previous mouse fMRI approaches using nociceptive heat stimuli, as demonstrated by the improved fidelity of spatial distribution and the substantial gain of the BOLD response. This is the first report of fMRI in wildtype mice with mild noxious contact heat stimuli (46 °C) that attains functional activation patterns closely resembling those observed in humans – a basic requirement for translational studies. Thermostimulation allows a controlled application of heat and cold stimuli above or below the pain threshold and thus permits research into clinically relevant forms of chronic pain, such as hyperalgesia and allodynia. Understanding the integrative nature of neural pain processing at a global level of brain circuits demands a reliable, non-invasive technique of high sensitivity and translational impact. Functional phenotyping based upon hemodynamic signatures of pain has the potential to utilize the broad spectrum of transgenic mouse models to elucidate the mechanisms that underlie thermal perception and pain. Thus, detailing the role of MABP as a potential confounder for hemodynamic readouts is paramount before nociceptive fMRI studies in mice can be interpreted unambiguously. The combination of mouse genetics and fMRI provides a promising tool for preclinical pain research and targeted drug development *en route* to new, effective therapies in particular for the relief of chronic pain^1^,^2^.

## Online Methods

### ROCKET design specification

All setup components were implemented in a custom-made modular mouse cradle (*ROdent Convective Keg-shaped Environment for Thermostimulation*, ROCKET), which conforms to the geometry of the cryogenically cooled RF coil (CryoProbe, Bruker Biospin, Ettlingen, Germany). The setup was implemented in a 9.4 Tesla, 20 cm bore MR system (Biospec 94/20, Bruker Biospin, Ettlingen, Germany).

The ROCKET consists of multiple modules that were designed as individual 3D CAD models using Autodesk Inventor Professional 2014 (Autodesk Inc, San Rafael, CA). The models were 3D-printed using ABS + material (Stratasys, Eden Prairie, MN) in a 3D printer (BST 1200es; Dimension Inc, Eden Prairie, MN). Printed modules were manually connected and modified by additional materials (see below). For an exploded-view sketch of ROCKET see Supplementary Figure S3 online.

#### Temperature regulated chamber

The chamber consists of three elements: a heated shell (divided in an upper and a lower section) and an underfloor heating system, each containing meander shaped channels supplied with warm water from a water circulation system (Supplementary Fig. S3 online). Each element was 3D-printed in two parts, an outer part comprising meander shaped cavities and an inner cover (Supplementary Fig. S4 online). The parts were joined together by coating them entirely with two-component epoxy to avoid water leakage. Additional coating with black paint (acrylic spray paint) was used to increase diffuse radiation. A plastic grid serving as support for a mouse is suspended between the chamber’s walls. The chamber covers the whole body length without touching the animal and thus represents a convection/radiation-based heating system. The water flow of both heating systems (floor and shell) is controlled by independent water baths, allowing individual thermal regulation for each system. The partitioning of the shell in an upper and a lower segment allows easy access to the animal’s hindpaw during experimental preparations by temporarily removing the upper shell, while the body continues to be warmed by the lower part and underfloor heating.

#### Hearing protection

Hearing protection was developed according to standards for human fMRI to protect the animal from acoustic noise generated by the MR gradient system^4^. Moldable wax earplugs were introduced into the auditory canal of the mouse using forceps and were gently removed after the fMRI session. Earmuffs were constructed by modifying the original ear bars of the CryoProbe using a pad sliced from a foam earplug (Ohropax Soft, Ohropax, Wehrheim, Germany), which was glued to a shim (plastic, 3 mm diameter, 0.5 mm thickness) (Supplementary Fig. S5 online). The customized earmuffs were sized to fit within the available space of the CryoProbe.

#### Thermal Stimulation System

The stimulation system comprises a miniaturized Peltier element (PE; size (14 × 14 × 2.5) mm^3^; PE-065-07-10, Telemeter Electronic, Kreuzlingen, Switzerland) driven by an electric current from a current source (P 1890, PeakTech, Ahrensburg, Germany), whose polarity is switched using a relay card (8× relay card 24 V/7 A, Conrad Electronic SE, Hirschau, Germany). The current source and relay card are controlled by a PC via an RS-232 interface. The PE is covered by a copper plate, which is used as a contact thermode. A fiber-optic temperature sensor (1 mm diameter; OTP-M, AccuSens, Opsens, Québec City, Canada) was incorporated in the thermode for feedback control of the PE via an in-house build software. The PE driving current is determined by a calibration (current vs. temperature lookup table) and adapted/corrected in real-time (every 200 ms) in a P-control algorithm, based on the actual thermode temperature. The PE heating performance is enhanced by a copper heat sink supplied with water of constant temperature (20 °C) from a water bath (Supplementary Fig. S6 online). Suggestions for size and shape variations of the thermode surface are outlined in Supplementary Figure S7 online.

*Contact pressure control.* The contact pressure control system consists of an elastic latex balloon, which is connected to a water-filled glass cylinder (Supplementary Fig. S6 online). By positioning the balloon between the hindpaw and a rigid resistance the balloon’s dilation provides a defined contact pressure of the paw onto the contact thermode. Pressure is controlled by gravity of the water in the cylinder, measured in millimeter water column above the balloon level. The balloon was made out of a finger cot sealed onto a luer-lock adapter using shrinkable tubing and highly fluid silicone to close all gaps. The stimulation system might be adapted for stimulation of the dorsal (hairy) side of the hindpaw (Supplementary Fig. S6d online).

### System tests and calibrations

#### Heating induced magnetic (B_0_) field homogeneity and signal change

To assess the potential effects of the stimulation device on the homogeneity of the magnetic field B_0_, we measured the local magnetic field shift in a homogeneous agarose phantom (2%) placed in the experimental setup. We acquired dual-echo gradient-echo scans (TE_1_ = 1.43 ms, TE_2_ = 5.71 ms) for no heating, baseline heating and maximum heating. Magnetic field maps were reconstructed in MATLAB (The MathWorks Inc, Natick, MA, USA). A Gaussian low pass filter was used to remove noise while maintaining the low spatial frequency field fluctuations induced by the current in the Peltier element. Differences between the low pass filtered field maps were calculated. The expected signal change due to the additional field gradient was calculated assuming a mono-exponential T_2_^*^ decay where the change in T_2_^*^ was estimated for each voxel based on the equation 1/T_2_^*^ = 1/T_2_ + γ|ΔB| with ΔB being the additional field gradient per voxel and γ the gyromagnetic ratio of the proton. T_2_ was assumed to be constant with regard to the field change. The expected signal change was examined for T_2_^*^ ranging from 1 ms to 100 ms. For comparison, the noise in the fMRI signal-time courses was expressed as the relative temporal standard deviation (rtSTD = temporal standard deviation/mean temporal signal intensity). The rtSTD is closely related to the temporal signal-to-noise ratio (tSNR = mean temporal signal intensity/temporal standard deviation). After ICA-filtering we extracted the signal time courses of six randomly chosen voxels in the S1 region. The relative tSTD and tSNR were calculated and then averaged over all voxels and all mice.

#### Thermo probes

For *in vivo* temperature measurements, six fiber optic temperature sensors were employed: a probe protectively covered in PTFE Teflon (diameter 1.5 mm) for rectal measurements (T1S-02-PW05-DC, Neoptix, Québec, Canada), a *bare probe* (T1S-02-B05-DC, Neoptix, Québec, Canada) optimized for surface measurements, and four cylindrical probes of 0.5 mm diameter (TC, Neoptix, Québec, Canada) for monitoring temperatures of the ambient air and the contact area of adjacent surfaces (like floor and skin). Temperature calibration measurements were performed outside the scanner using a pre-calibrated thermometer (P700-Universal-Thermometer, Pt100 sensor, Dostmann electronic, Wertheim-Reicholzheim, Germany) in oil media. Due to interferences with the magnetic field the fiber probes have a consistent offset of −4.7 K inside the 9.4 T MR scanner. This offset was corrected during post-processing. The relative error of the thermo probes was ±0.2 K.

#### Thermal measurement of the CryoProbe surface

The CryoProbe thermal shield was adjusted to either 38 °C or 48 °C. The RF coil was removed from the scanner and the CryoProbe surface was measured along its full length using the *bare probe*. The measurements were conducted at an environmental temperature of 20 °C.

### Animal experiments

Animal experiments were carried out in accordance with the guidelines provided and approved by the Animal Welfare Department of the *Landesamt für Gesundheit und Soziales* (LaGeSo) Berlin (Berlin State Office of Health and Social Affairs). All mice were housed in groups of 4–6 animals in cages with nesting material, mouse lodges and open access to water and feed, at 24 °C with a 12 h/12 h circadian cycle.

#### Temperature measurements of awake mice

Nine male C57BL/6 N mice (weight 25–32 g) were randomly selected from different cages. Skin temperatures were measured using the *bare probe* for dorsal and ventral trunk (both cranial and caudal) and scalp. After taking an animal from its cage temperature measurements were carried out immediately while the animal was held in the air (20 °C room temperature). In the same manner thermal map images were acquired using an infrared camera (Ti25 Thermal Imager, Fluke, Everett, WA, USA).

#### Temperature measurements of anesthetized mice

Sixteen male C57BL/6 N mice (weight 25–32 g) were anesthetized using isoflurane (induction: 2–3% isoflurane, maintenance: 1.3% isoflurane) in an 80%/20% air/oxygen mixture. To prevent an initial drop in body temperature, induction of anesthesia occurred in a pre-warmed, red-transparent plastic tube. Anesthetized mice were directly transferred to one of the following MR mouse cradles: a cradle designed for a conventional 4-element radiofrequency (RF) mouse head coil array (CONV; n = 5) (model no.: 1 P T11457, Bruker BioSpin, Ettlingen, Germany), the mouse cradle of the CryoProbe (CRYO; n = 5) (Bruker BioSpin, Ettlingen, Germany) and the customized ROCKET cradle described above (n = 6). The cradles were inserted into the MR bore and water bath temperature was adapted to maintain a stable, physiological rectal temperature of 36.3 °C ± 0.5 °C in each setup. Temperatures were monitored simultaneously at various locations and temporal averages were calculated for each animal.

Cylindrical probes were used to measure ambient air temperature and temperatures at the interface of the ventral trunk and floor. The *bare probe* was used for direct surface measurements of the dorsal trunk. The scalp temperature was assessed using a cylindrical probe positioned between the scalp and an insulating sheet (polyethylene foam) covering the RF coil’s surface (Fig. 1b, bottom panel). The thermo probe itself (0.5 mm diameter) creates spacing between the rigid scull and the coil surface. The soft foam sheet was introduced to fill the lateral gaps and thus prevent thermal influence of the ambient air on the temperature sensor. For the CONV setup, the cranial part of the ventral trunk-floor interface (front) was measured because the CONV mouse bed consists of two separate segments, impeding heat transfer from the floor heating system to the frontal region (Fig. 1a).

#### Evaluation of the stimulation temperature

Thermode surface temperature measurements were performed using the *bare probe*. Thermode-skin-interface temperature was assessed using a cylindrical fiber optic probe (Fig. 2c). To account for impaired heat transfer at the measuring site, thermal conductance paste was applied between the paw and the thermode surface. This was necessary since the cylindrical probe itself hinders sufficient heat transfer for tissue concealed by the probe during the rapid transition times.

#### Contact pressure adjustment

Thermode contact pressure was determined by visual inspection of the paw in an initial study. To exclude potential obstruction of blood perfusion no change of skin color should occur when removing the thermode from the paw. The water column level, which was consistently adjusted for the feasibility study, might be adapted for different setups in respect to size and elasticity of the balloon and the spacing to the thermode (Supplementary Fig. S6c online).

#### Blood gas analysis

To ensure physiological conditions under mechanical ventilation the blood gas levels (pCO_2_) were determined in seven male C57BL/6 N mice (weight 25–30 g) using a transcutaneous blood gas analyzer (TCM4 equipped with baby clips, Radiometer, Copenhagen, Denmark) at the shaved upper hind limb.

### Feasibility study

#### Animal preparation

Six male C57BL/6 N mice (weight 23–28 g) were positioned in the customized mouse bed ROCKET and studied using heat stimulation. Mice were anesthetized using isoflurane (induction: 2–3% isoflurane, maintenance: 1.3% isoflurane, in an 80%/20% air/oxygen mixture), endotracheally intubated and mechanically ventilated to ensure stable physiology (80 bpm, 25% inhalation, 75% expiration; MR-1 ventilator, CWE incorporated, Ardmore, USA). The head was stereotactically fixed using a tooth bar and customized earmuffs (see above). An eye gel was used to prevent the eyes from becoming dry during the experiment. Animal preparation was carried out within a plastic box that enclosed the entire frontal part of the mouse bed, constantly warmed by influx of air at 36 °C. After preparation the thermal chamber of the ROCKET was closed and the ROCKET was immediately transferred into the magnet. For the fMRI acquisitions anesthesia was reduced to 1.0% isoflurane. Functional scans were started 60 min after induction and 10 min after reduction of isoflurane. To prevent motion the animals were immobilized prior to fMRI using a muscular blocking agent (pancuronium bromide, 1 mg/kg; Sigma-Aldrich, Steinheim, Germany).

#### Thermal and device settings

Floor and shell temperatures were adjusted to stabilize a rectal temperature of 36.3 ± 0.5 °C while simultaneously maintaining dorsal and ventral trunk temperatures at 36.3 ± 0.5 °C. Temperature probes were used as described above for continuous monitoring of rectal temperature, as well as the skin temperature of the dorsal trunk, ventral trunk, scalp, the head space and the ambient temperature within the chamber.

#### Temperature stimulation paradigm

Thermostimulation was performed at the right plantar hindpaw using paradigms of 35.5 °C baseline, with transition rates of 4 °C/s and thermode core peak temperatures of 48 °C. Stimuli were repeated 4 times with an interstimulus interval of 90 seconds. Thermode-skin-interface temperature was not monitored to provide unhindered heat transfer between thermode and paw.

#### Magnetic resonance imaging (MRI)

MRI experiments were performed on a 9.4 Tesla Bruker small bore animal MR system (BioSpec 94/20, Bruker BioSpin, Ettlingen, Germany). A cryogenic quadrature RF probe (CryoProbe, Bruker BioSpin, Ettlingen, Germany) was used for signal transmission and reception. The CryoProbe thermal shield was adjusted to 48 °C. During all imaging sessions a thin insulating sheet was positioned between scalp and coil surface. Scalp temperatures were constantly monitored to lie within 36 °C ± 0.5 °C.

Pilot images and high spatial resolution sagittal T_2_-weighted images were used to position the structural reference scan, which consisted of 21 axial T_2_-weighted slices (RARE, echo train length 16, TR 3344 ms, TE 49.3 ms, FOV (24 × 12) mm^2^, matrix 192 × 96, spatial in-plane resolution (125 × 125) μm^2^, slice thickness 500 μm, slice spacing 600 μm) covering the entire mouse brain. Prior to fMRI magnetic field homogeneity was improved by voxel-based shimming using the MAPSHIM technique, which calculates the 1^st^ and 2^nd^ order shims for a defined volume of interest (shim voxel) based on an acquired B_0_ field map. The geometry of the shim voxel was set such that it covers the dorsal part of the brain excluding the air filled anterior cavities. For T_2_^*^-weighted fMRI stacks of 11 axial slices were acquired using gradient echo echoplanar imaging (GE-EPI, TR 2500 ms, TE 11.0 ms, flip angle 90°, bandwidth 300 kHz, FOV (24 × 12) mm^2^, matrix 90 × 60, spatial in-plane resolution (267 × 200) μm^2^, slice thickness 500 μm, slice spacing 600 μm). The acquisition matrix was zero-filled to 128 × 64 yielding a voxel size of (188 × 188 × 600) μm^3^. A temporal resolution of 2.5 s was applied together with 304 repetitions resulting in a total duration of 12 min and 40 sec. To allow for post-acquisition distortion correction of the functional scans another 3D B_0_-map was acquired with the same geometry used for the functional scans, but in 3D mode using a matrix size of 192 × 96 × 22 and a FOV of (24 × 12 × 13.2) mm^2^. A 3D volume of high spatial resolution T_2_-weighted images (3D RARE, echo train length 16, TR 2000 ms, TE 46 ms, FOV (13 × 13 × 21) mm^3^, matrix 192 × 192 × 60, resolution (68 × 68 × 350) μm^3^) was acquired separately to serve as anatomical background for the functional activation maps.

#### Monitoring of mean arterial blood pressure and heart rate

Animal preparation was conducted as described above. The left femoral artery of six male C57BL/6 N mice (weight 23–28 g) was cannulated to allow for continuous monitoring of mean arterial blood pressure (MABP). Mice were positioned in the customized mouse cradle ROCKET to study MABP and heart rate in response to subsequent heat stimuli. Animal preparation and experiments were carried out within a plastic box that enclosed the entire frontal part of the mouse bed, constantly warmed by influx of air at 36 °C. For heat stimulation tasks anesthesia was reduced to 1.0% isoflurane. Stimulation paradigms started 60 min after induction and 10 min after reduction of isoflurane. Animals were immobilized prior to heat stimulation tasks by intravenous injection of a muscular blocking agent (pancuronium bromide, 1 mg/kg; Sigma-Aldrich, Steinheim, Germany).

### Data processing and analysis

MRI data were processed by an in-house built processing pipeline (written in UNIX shell scripts bash and zsh). All analyzing and processing tools employed are part of the FMRIB’s Software Library^87^ (FSL, Oxford, UK) if not stated otherwise. Prior to image processing MR data were converted to NIFTI-format and scaled to human dimensions (x,y,z-voxel size ×20) to better meet the requirements of human fMRI data processing and display.

#### fMRI data processing

Functional time-series were motion corrected (MCFLIRT). Signal time courses were decomposed in 30 independent components using independent component analysis (MELODIC). From the set of independent signal components subcomponents were identified that represent signals of no interest, such as global intensity drift, head motion and respiration induced periodic field distortions. The exclusion criteria were: signals predominantly present at the brain borders, signal frequencies that are very low (<10 cycles; drift) or around the respiratory frequency (respiratory motion /induced field distortions)^83^,^88^. These components were removed from the signal in correlated voxels (Supplementary Fig. S8 online).

#### Distortion correction

EPI distortion correction was applied to the denoised functional data via FUGUE to account for magnetic field inhomogeneity induced image distortions. To provide compatible data to FUGUE, the acquired 3D B_0_ field map (Bruker 2dseq file) was scaled (by multiplication with the VisuCoreDataSlope parameter) to obtain the field shift in Hz. Subsequent multiplication with 2π converted Hz into radians. The field map 3D volume was converted to NIFTI and cropped in z direction to fit the geometry of the functional scans.

#### Anatomical reference in Waxholm Space (WHS)

The anatomical reference was created based on a 15 μm average C57BL/6 mouse model in Waxholm space (http://www.imaging.org.au/AMBMC/ Model)^89^. The symmetrical brain volume was downscaled to a matrix size of 85 × 62 × 69 voxels. Voxel sizes were modified to 2.4 × 2.4 × 5.7 mm, which translates to an actual voxel size of 120 × 120 × 285 microns. Voxels outside the brain were set to zero.

#### Normalization in Waxholm Space

Structural image volumes were filtered using a *spatially adaptive non-local means* (SANLM) filter^90^ to consider spatially varying noise levels, which is available as part of the VBM8 toolbox (http://dbm.neuro.uni-jena.de/vbm8/) of SPM8 (http://www.fil.ion.ucl.ac.uk/spm/). Scull stripping was performed using the *brain extraction tool* (BET). Normalization to the anatomical reference in WHS^30^ was applied using non-linear diffeomorphic image registration (12 degrees of freedom) by explicit B-spline regularization^85^, which is part of the Avant’s Normalisation Tool “ANTs”^86^. Diffeomorphic image registration techniques^91^ were recently introduced in mouse fMRI^92^,^93^,^94^. Distortion corrected functional time-series were scaled to the dimensions of the structural image volumes and spatial transformations and warps (resulting from the registration step) were applied prior to conjunction analysis.

#### Spatial smoothing

Smoothing was applied with a FWHM of 6 mm (which accords to 300 μm) (SUSAN) prior to statistical analysis using FEAT.

#### Statistical analysis

FMRI data processing was carried out using FEAT (FMRI Expert Analysis Tool) Version 6.00, part of FSL. Data from the initial 4 time points of a time-series were discarded to exclude hardware and saturation related artifacts. 4D data was high pass filtered with a cutoff of 140 seconds equaling one stimulus duration plus the initial baseline period. General linear model analysis was performed by convolving a binary (0 = off, 1 = on) regressor with double-gamma functions and their temporal derivatives. The onset of the regressor was set 6 seconds after onset of the stimulus, when thermode-skin-interface-temperature exceeded 46 °C. Durations of 35 seconds were chosen in order to model for the slow decline of the hemodynamic response curve observed in our initial experiments, which was also previously reported for mouse fMRI of electrostimulation and likely caused by vasodilatatory effects of isoflurane^9^. Motion parameters were not included into the statistical analysis, but directly removed from the data by motion correction (see above). Remaining head motion (e.g. rhythmic ventilation-induced motion) was eliminated by ICA (for details see Supplementary Fig. S8 online).

#### Second level analysis

Higher-level modeling was conducted using full FMRIB’s local analysis of mixed effects (FLAME, stages 1 + 2). Z (gaussianised T/F) statistic images were thresholded using GRF-theory-based maximum height thresholding with a (family wise error (FWE) corrected and uncorrected) significance threshold of p = 0.05^95^. Resulting group z-statistic maps were superimposed on a high-resolution anatomical 3D image volume, which was normalized in WHS.

#### Anatomical classification

Spatial overlaps of significant clusters with anatomical brain regions were identified by applying anatomical atlas masks^96^,^97^ onto the group z-statistic map (spatially normalized in WHS). Atlas masks are available from the webpage of the Australian Mouse Brain Mapping Consortium (http://www.imaging.org.au/AMBMC) in the same coordinate space as the reference model^89^ applied for spatial normalization. Thalamic regions were specified using the Allen Mouse Brain Atlas provided in WHS (atlas.brain-map.org).

#### BOLD signal time courses

Signal time courses were created for significant voxel clusters in specified anatomical regions at p < 0.05 (FWE). Region of interest (ROI) masks were created by binerizing significant voxel clusters, which were then multiplied with atlas masks for specified anatomical brain regions. The resulting ROI masks were applied to the functional timeseries and used to extract intensity time courses for each subject. Signal change (%) was calculated ((signal * 100/baseline) − 100) and averaged over all animals.

## Additional Information

**How to cite this article**: Reimann, H. M. *et al.* Normothermic Mouse Functional MRI of Acute Focal Thermostimulation for Probing Nociception. *Sci. Rep.* **6**, 17230; doi: 10.1038/srep17230 (2016).

## Supplementary Material

Supplementary Information
