# Supplementary material for: Normothermic Mouse Functional MRI of Acute Focal Thermostimulation for Probing Nociception
Source: Sci Rep. 2016 Jan 29;6:17230. doi: 10.1038/srep17230 (PMC4731789; doi:10.1038/srep17230)
Supplement: Supplementary Information [file srep17230-s1.pdf]

Supplementary Figures

Normothermic Mouse Functional MRI of Acute Focal Thermostimulation for Probing Nociception

Henning Matthias Reimann[1], Jan Hentschel[1], Jaroslav Marek[1], Till Huelnhagen[1], Mihail Todiras[2], Stefanie Kox[1], Sonia Waiczies[1], Russ Hodge[2], Michael Bader[2], Andreas Pohlmann[1], Thoralf Niendorf[1,3§]

[1] Berlin Ultrahigh Field Facility (B.U.F.F.), Max Delbrueck Center for Molecular Medicine, Berlin, Germany

[2] Max Delbrueck Center for Molecular Medicine, Berlin, Germany

[3] Experimental and Clinical Research Center, a joint cooperation between the Charité Medical Faculty and the Max Delbrueck Center for Molecular Medicine, Berlin, Germany

§ Corresponding Author:

Prof. Dr. Thoralf Niendorf

Berlin Ultrahigh Field Facility (B.U.F.F.)

Max Delbrueck Center for Molecular Medicine,

Robert Roessle Str. 10,

13125 Berlin, Germany

Tel: 0049 30 9406 4504,

Fax: 0049 30 9406 4517

Email: Thoralf.Niendorf@mdc-berlin.de

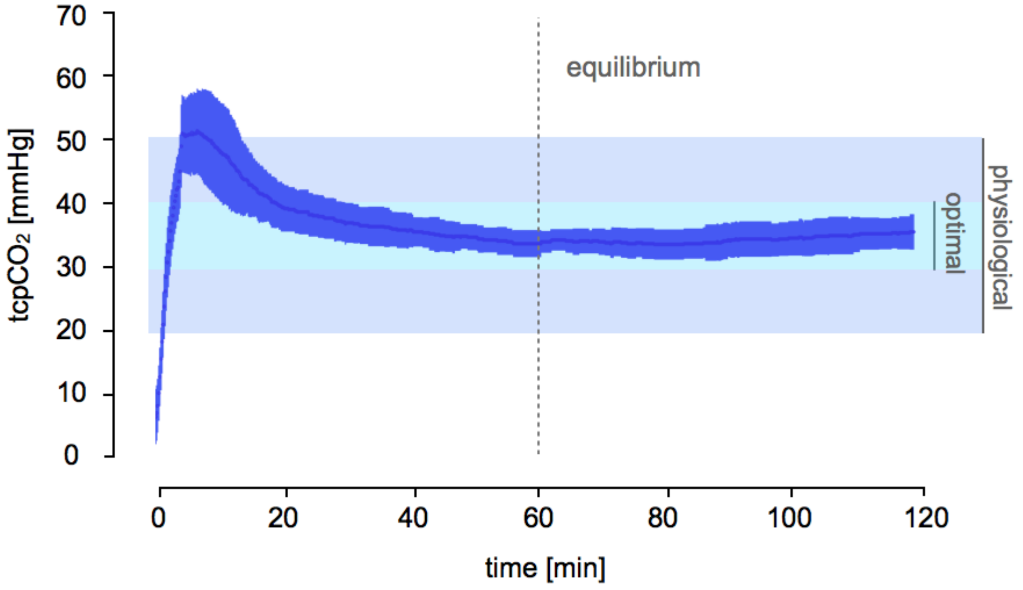

Supplementary Fig. S1. Time course of transcutaneous CO_2 levels ($tcpCO_2$) during mechanical ventilation of mice (n = 7) using a non-invasive blood gas monitoring device. After equilibrium is reached at the measuring electrode, CO_2 values were assessed in an optimal, physiological range[22] for all tested animals (mean (s.d.)).

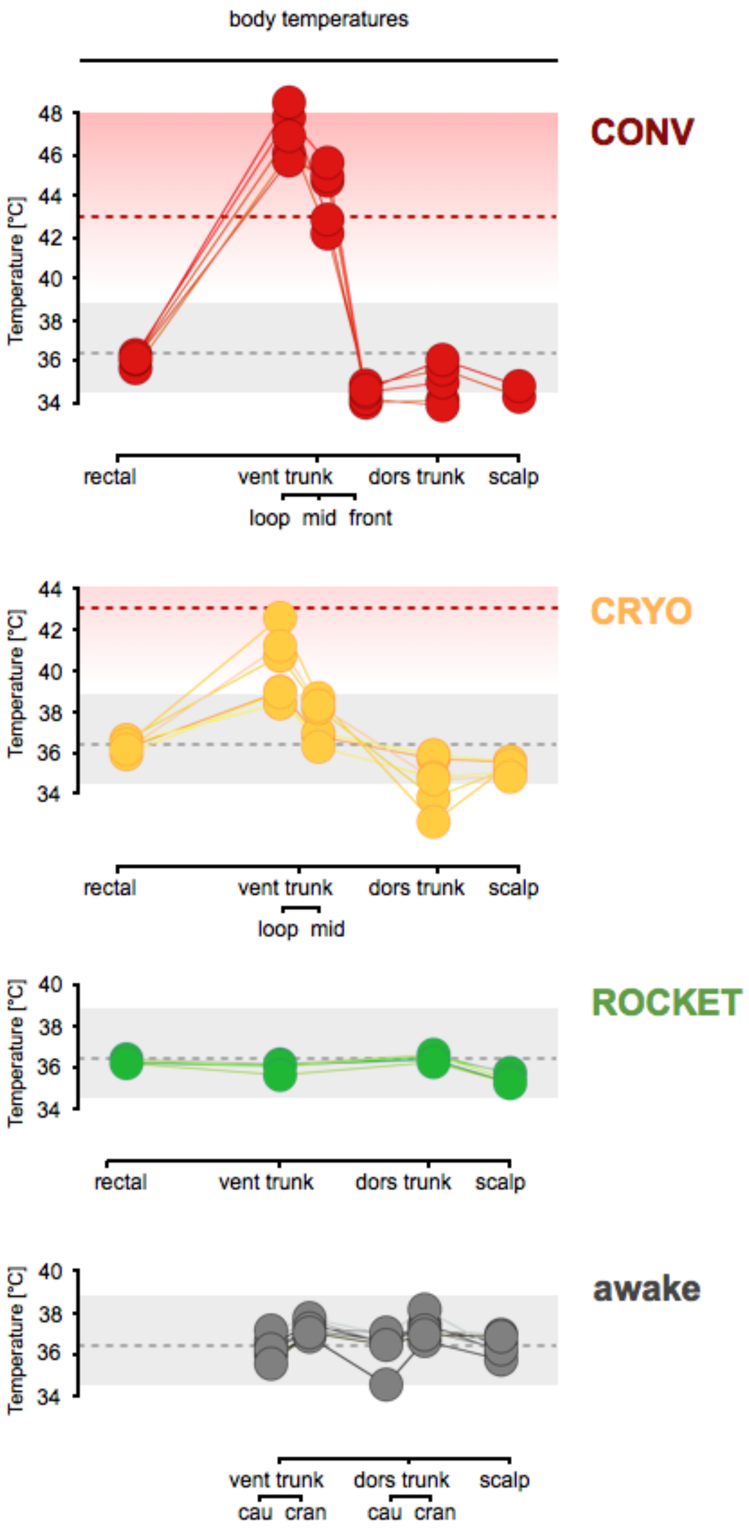

Supplementary Fig. S2. Detailed plot of body temperature measurements. Anesthetized mice were placed in three different fMRI setups: A conventional setup (CONV; n = 5), the CryoProbe setup (CRYO; n = 5) and the ROCKET setup (n = 6). Temperatures are plotted for the rectum, ventral trunk (above heating loops, at mid or front position (see **Fig. 1a**)), dorsal trunk and scalp (see **Fig. 1b**). Body surface temperatures of awake animals are plotted as a reference. Temperatures were assessed for ventral and dorsal trunk (cranial and caudal), and scalp. Trunk temperatures of cranial and caudal sites were averaged for presentation in the Results section and Figure 1c. The temporal mean of each temperature measurement is plotted as a single dot. Measurements for individual animals are connected by lines to guide the eye.

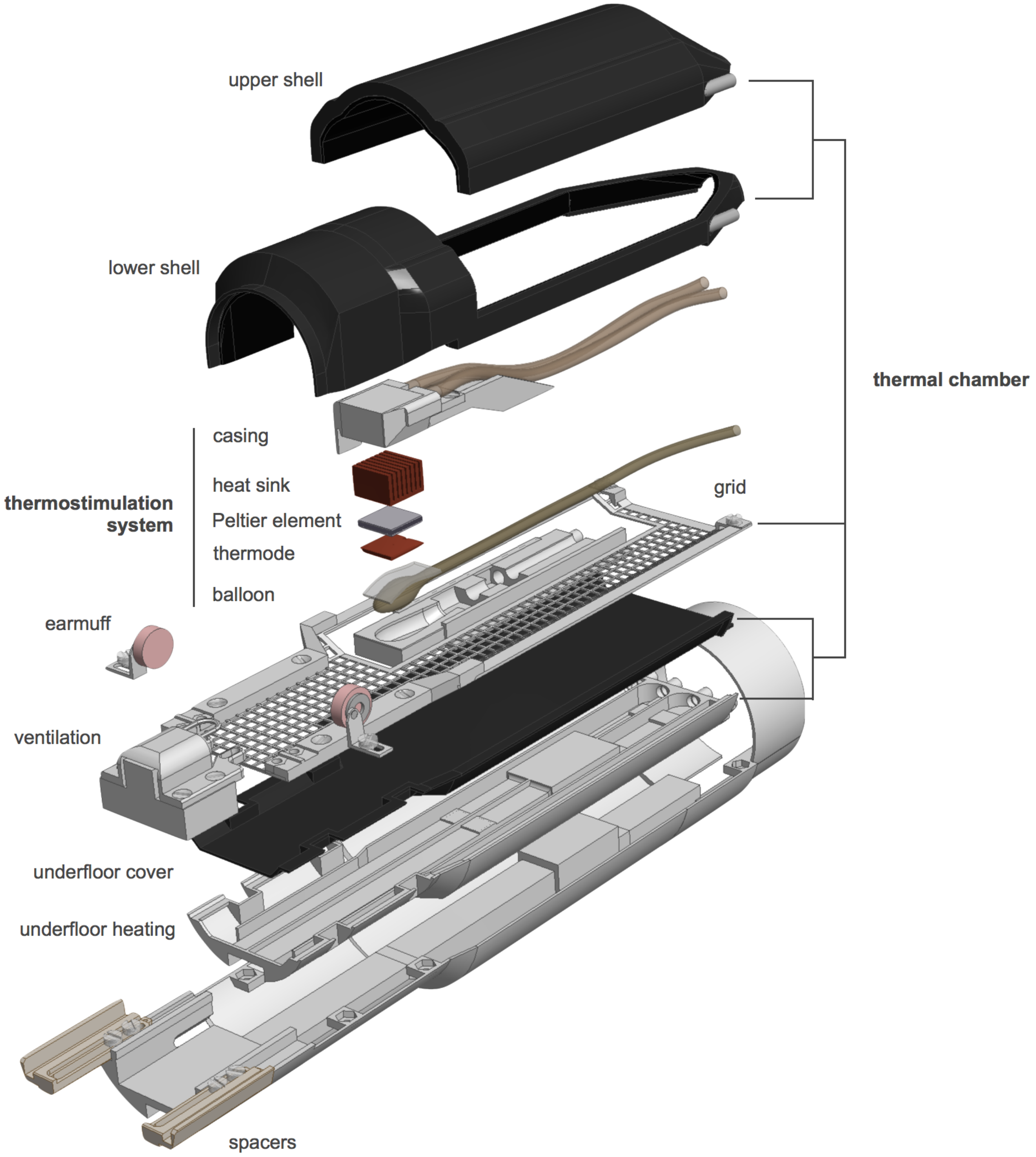

Supplementary Fig. S3. Exploded-view sketch of the ROCKET. The *thermal chamber* consists of a *shell* above and a *floor heating system* below the animal enclosing its entire body with exception of the head (**Fig. 3**). The *shell* is subdivided into an *upper* and *lower* segment allowing for easy access to the animal's paw without removing the entire shell. Each element comprises meander shaped channels for warm water supply (**Supplementary Fig. S2**). The animal is positioned on a plastic *grid* to prevent direct contact with the heated components and receives heat energy by convection and radiation (**Fig. 1b**). The thermal stimulation system is based on a feedback controlled Peltier element (*PE*), a water-cooled *heat sink* encapsulated in a *casing*, and a hydraulic, inflatable *balloon* positioned below the paw to apply mild contact pressure towards the *thermode* (**Supplementary Fig. S3**). *Earmuffs* are employed for hearing protection (**Supplementary Fig. S5**). A *ventilation* block, which comprises a tooth bar to stabilize the rostral head, accommodates the respiratory junction of the endotracheal tube.

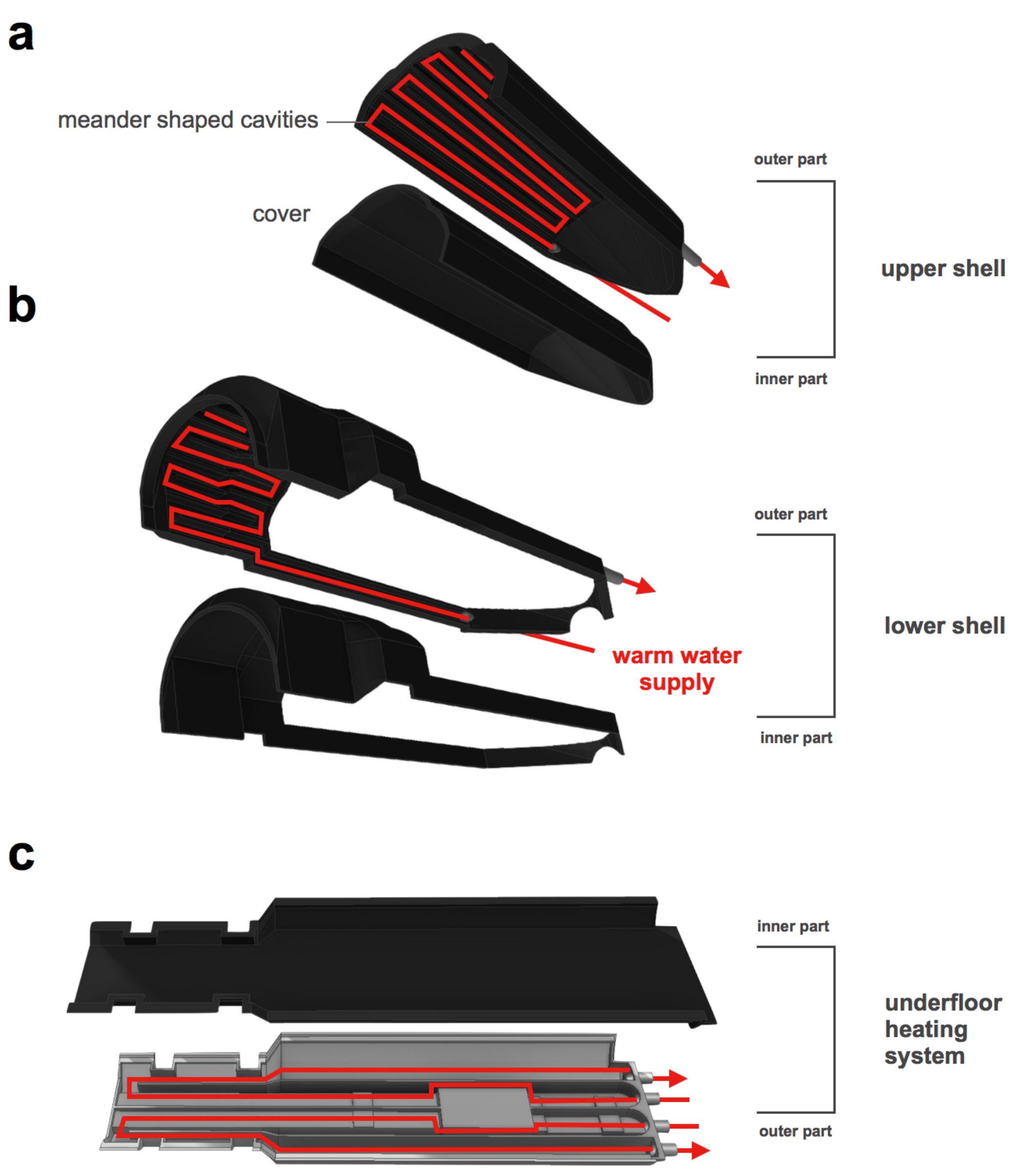

Supplementary Fig. S4. Temperature regulated multi-element chamber. The sketch illustrates the water flow through meander shaped channels in upper (**a**) and lower shell (**b**) and in the underfloor heating system (**c**). Each module consists of two segments, which are connected and sealed to avoid water leakage.

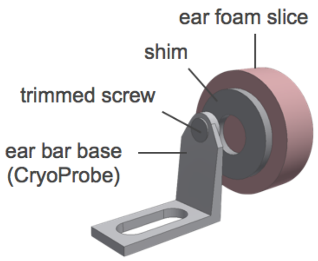

Supplementary Fig. S5. Hearing protection. Customized earmuff, composed from a conventional mouse MRI ear bar (for the Bruker CryoProbe). An assembly of a slice of a memory foam earplug, a plastic shim and a trimmed plastic screw is attached. The lateral extent of the earmuffs was adapted to fit the limited space within the CryoProbe. To improve hearing protection moldable wax earplugs were introduced into the auditory channel.

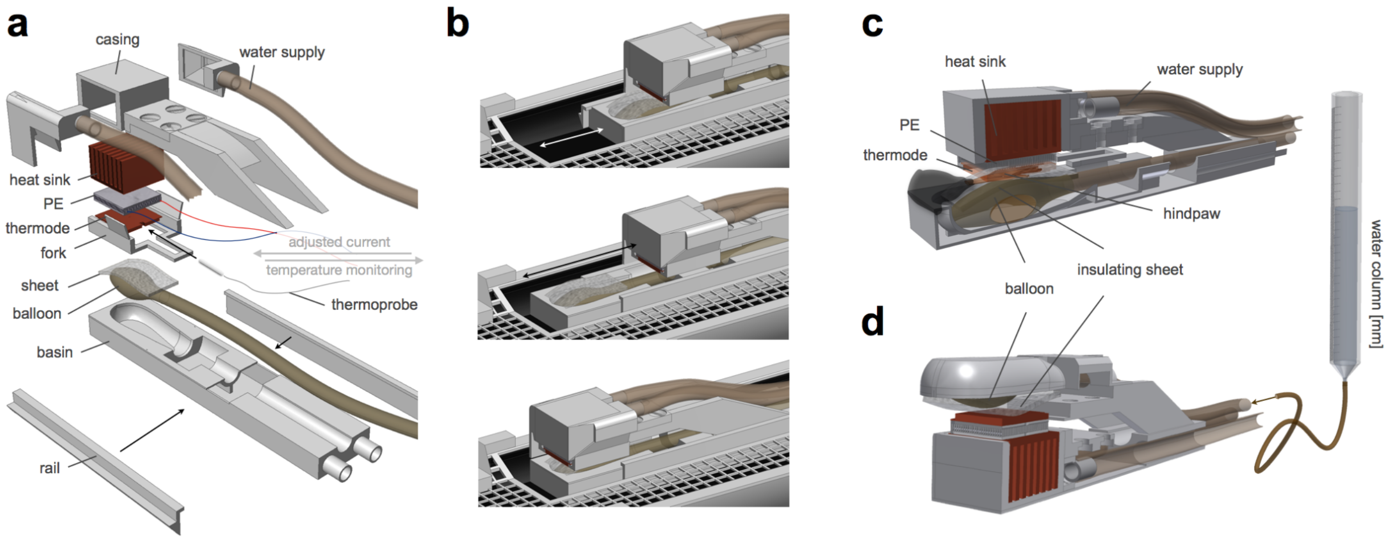

Supplementary Fig. S6. Thermal stimulation system. (**a**) An exploded-view sketch illustrates all components applied for thermal stimulation and contact pressure control of the plantar hindpaw. The stimulation assembly includes the Peltier element (*PE*) supported by a water-cooled copper block (*heat sink*), a copper *thermode* (stabilized by a plastic *fork*) covering the entire *PE*, and a fiber optical *thermoprobe*, which is incorporated in the *thermode*. The actual temperature is adapted (via a feedback-control algorithm) by adjusting the PE driving current. The contact pressure assembly involves an inflatable *balloon* connected to a water-filled cylinder. The cylinder's filling level (in mm *water column* above the paw) determines the balloon's dilation (c,d, right panel). The balloon resides within a suited *basin* to restrict its lateral extension. An *insulating sheet* of silicone foam impedes thermal influence from the balloon to the paw's backside. (**b**) Lateral rails permit the individual positioning of the stimulation system to account for varying body lengths of the tested animals, and allow a comfortable positioning of the paw. (**c**) Assembly of the plantar thermostimulation system shows the positioning of the hindpaw. Panel (**d**) introduces an alternative design for dorsal paw stimulation.

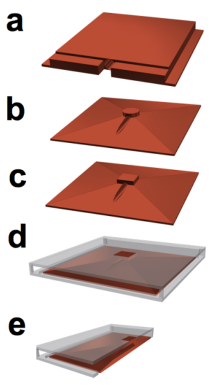

Supplementary Fig. S7. Suggested shape and size variations of the contact thermode. The copper thermode used in the feasibility study (**a**) comprises an almost full size surface (10x12 mm) to recruit multiple receptive fields of heat sensitive fibers. For smaller stimulation areas it is recommended not to use smaller PE. To maintain heating/cooling performance a protruding area in the thermode‘s centre of desirable shape and size might be used, e.g. a 3x3 mm cylinder (**b**) or rectangle (**c**). Smooth angled pyramid shapes facilitate the heat transfer from the foundation to the contact area. The incorporated thermoprobe in the foundation (for feedback-control) might be located close to the contact area to ensure faster reaction times to the actual stimulation temperature. When smaller contact areas are applied, a solid platform around the contact area permits an even contact pressure to the entire paw (**d**,**e**).

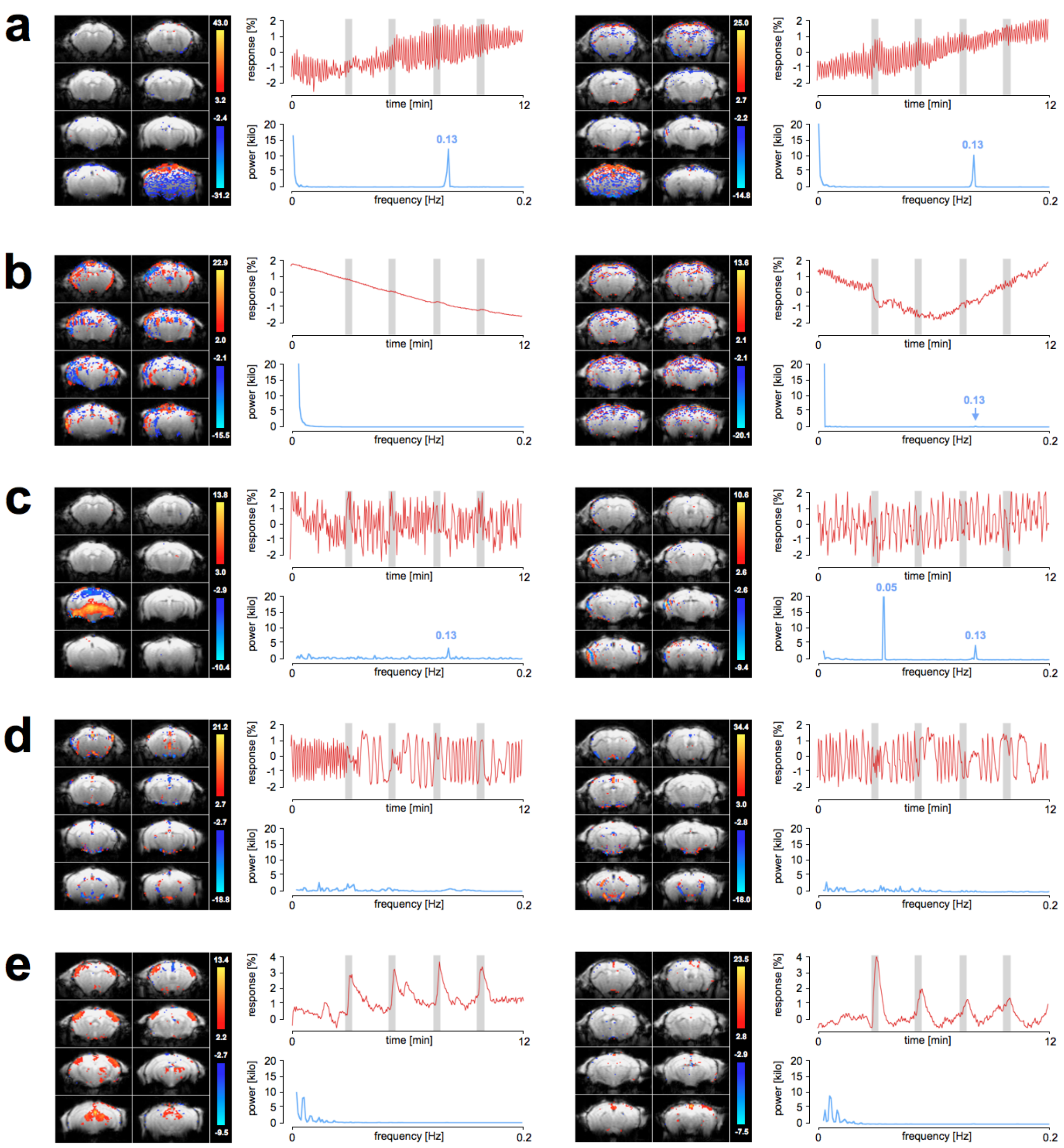

Supplementary Fig. S8. Examples of independent signal components (ICs) detected by independent component analysis (ICA) of the fMRI data (heat stimulation). ICA was performed as part of the preprocessing procedure for single subjects after motion correction (see Online Methods). Detected ICs could be classified into five categories: (**a**) rhythmic /periodic frequencies at 0.133 Hz, (**b**) low frequency signal drifts, (**c**) periodic noise, (**d**) noise with no obvious periodic contribution, and (**e**) stimulus related signal changes.

Categories (**a**)-(**c**) were considered nuisance signals and removed from the data before the statistical analysis. These are either signal drifts (very low frequencies) or periodic signals of no interest with one or several dominating distinct frequencies (e.g. 0.133 Hz). Periodic signals at 0.133 Hz can be explained by remaining rhythmic ventilation-induced B_0 shifts or motion at 80 bpm: the temporal resolution of the fMRI was 2.5 seconds, resulting in a sampling rate of 0.4 Hz. This is lower than the respiration induced signal (1.33 Hz) and violates the Nyquist theorem, which results in aliasing at a frequency of 0.133 Hz.

	a	b	c	d	e	rem	total
mouse #1	7	2	4	11	6	**13**	30
mouse #2	9	0	2	10	9	**11**	30
mouse #3	7	1	3	10	9	**11**	30
mouse #4	5	1	12	5	7	**18**	30
mouse #5	6	1	2	15	6	**9**	30
mouse #6	6	1	7	8	8	**14**	30

Supplementary Table 1. Number of independent signal components (ICs) for each subject of the fMRI dataset (heat stimulation) classified into five categories: (**a**) rhythmic /periodic frequencies at 0.133 Hz, (**b**) low frequency signal drifts, (**c**) periodic noise, (**d**) noise with no obvious periodic contribution, and (**e**) stimulus related signal changes. For details see Supplementary Figure S8. From a preset of 30 components ICs of category a-c were removed. The total number of removed ICs is listed for each subject (rem).
